# Moderating role of mindfulness and social support in the relationship between fertility pressure and fertility quality of life in Chinese infertile men

**DOI:** 10.1371/journal.pone.0321877

**Published:** 2025-06-10

**Authors:** Lihuan Zhi, Jumanali Mireyi, Abulizi Maierhaba, Hua Xu, Lijuan He, Jiangabieke Lizha

**Affiliations:** 1 School of Public Health, Xinjiang Medical University, Urumqi, Xinjiang, China; 2 The First Affiliated Hospital of Xinjiang Medical University, Urumqi, Xinjiang, China; Dicle University, TÜRKIYE

## Abstract

Men with infertility are susceptible to fertility pressure, thus affecting their fertility quality of life. To develop fertility-pressure resilience interventions, we investigated whether mindfulness and social support could buffer the association between perceived fertility pressure and fertility quality of life in Chinese infertile men. In this cross-sectional study, Chinese men with infertility who visited the clinic for semen examination completed the Fertility Pressure Inventory, Mindful Attention Awareness Scale, Social Support Scale, and Fertility Quality of Life Scale online. In total, 469 Chinese infertile men (mean age: 32 years) were recruited, of whom 414 (88.3%) reported no childbearing history or high fertility stress. Analysis using generalised linear models revealed that social support had a moderating effect on the association between reproductive stress and reproductive quality of life (*F* = 11.006, *P* = 0.001), and between the core (*F* = 9.063, *P* = 0.003) and treatment (*F* = 9.383, *P* = 0.002) subdimensions of reproductive quality of life and reproductive stress. Among men with high social support scores, the association between reproductive stress and reproductive quality of life (*t* = −3.146, *P* = 0.002) and the core subdimension (*t* = −3.333, *P* = 0.001) was significant, whereas among men with low social support scores, the association between reproductive stress and reproductive quality of life (*t* = 0.906, *P* = 0.365) and the core subdimension (*t* = 0.266, *P* = 0.790) was not significant. Mindfulness did not significantly regulate fertility stress and reproductive quality of life(*F* = 1.528, *P* = 0.217), its core (*F* = 1.406, *P* = 0.236) and treatment (*F* = 1.026, *P* = 0.312) subdimensions. Higher social support levels attenuated the negative association between fertility stress and reproductive quality of life (including core dimensions) in infertile men. Experimental studies are needed to determine whether social support is a protective factor.

## Introduction

Infertility is a disorder of the men reproductive system defined by the World Health Organization as a couple that has not become pregnant for one year or more after having unprotected sex [1]. It is estimated that one in six people of childbearing age worldwide is infertile, with men factors accounting for approximately half of cases [2]. men infertility is an important factor affecting the global population development. Studies have shown that infertility is related to many factors, such as genetic and environmental factors and lifestyle [3]. However, patients usually experience great physical pain when undergoing treatment, and the treatment success rate is often low [4]. Although infertility does not affect normal life, it has a profound impact on their families and quality of life [5]. Therefore, reducing the social and public health burden of men infertility requires identifying the causes of prevention, especially the modifiable risk factors.

Fertility stress is a common factor associated with men reproductive quality of life [6]. Fertility pressure comes mainly from three aspects: oneself, family, and society [7]. In the traditional Chinese culture, having children is considered the family mission. A man who is infertile due to reproductive health problems has a great tendency toward anxiety and depression, although this degree is lower than that in femen infertile patients [8]. Assisted reproductive technology is the most important and effective method for infertility, but due to the long treatment cycle, high cost, and uncertain treatment outcomes, patients experience a heavy psychological burden and mental pressure, which affects their treatment compliance and seriously affects their mental health and quality of life [9]. Some scholars found that the fertility pressure score of the infertility group was significantly higher than that of the control group, and the quality-of-life score was significantly negatively correlated with the fertility pressure score. The higher the fertility pressure, the worse the quality of life of patients [10]. Therefore, fertility pressure should be reduced as an entry point, and humanised strategies should be provided for patients.

Stress in men with infertility is closely related to social support. Social support refers to the assistance, resources and emotional support that men infertility patients receive from their partners, family, friends, healthcare team and social network that aims to relieve their psychological stress due to infertility, improving treatment adherence, enhancing coping skills, and improving overall quality of life [11]. Some scholars have confirmed that the total social support score for men with infertility is negatively correlated with the total fertility pressure score. This indicates that positive social support can alleviate fertility pressure [12]; however, it remains unclear whether interpersonal and intrapersonal psychological resources protect men from the negative effects of reproductive stress on quality of life. Specifically, we examined whether social support can be used as a protective factor against a decline in reproductive quality of life in groups with reproductive stress.

In addition, as a positive psychological quality, mindfulness refers to when men with infertility can reduce stress, improve emotional regulation and increase mental resilience to infertility-related challenges by consciously paying attention to the present moment, and becoming aware of and accepting their emotions, thoughts and physical sensations in relation to fertility issues in a non-judgemental manner [13]. men infertility is not only a physical disease, but it can also have a serious impact on the mental health of the individual. Van Eickels D’s [14] study found that infertility can lead to feelings of low self-esteem, helplessness, anxiety and even depression, while Cserepes RE’s [15] study suggests that the disease can cause men to feel that they are unable to fulfil traditional gender roles, which can lead to a decline in self-esteem. Recently, mindfulness interventions have been shown to improve patients’ negative emotions, self-efficacy, and physical and mental health [16]. Kang et al. [17] conducted a 4-week mindfulness intervention on 73 infertile women and found that the level of mindfulness improved, and the dimension of childless lifestyle had a significant effect compared to that before the intervention. However, whether mindfulness modulates the association between fertility stress and reproductive quality of life remains unclear. Therefore, this study introduces the concept of mindfulness to investigate whether it can act as a moderator to buffer the negative effects of fertility stress on reproductive quality of life.

Based on the above findings, we hypothesised that social support and mindfulness may play an important role in the relationship between fertility pressure and the reproductive quality of life of men with infertility. In addition, having higher psychological resources and social support may reduce the negative impact of higher fertility pressure on the reproductive quality of life of such men. Therefore, to test this hypothesis, this study explored whether social support and mindfulness play a moderating role in the association between reproductive stress and reproductive quality of life in men with infertility, and providing information for designing future psychosocial interventions.

## Materials and methods

### Participants

In this cross-sectional study, 469 men infertility patients who visited the prenatal diagnostic unit of a tertiary hospital in Xinjiang for semen examination were screened between 2021–2024. The researcher had allowed the patients to fill in an anonymous electronic questionnaire during the time between their visit and delivering their semen samples to the semen analysis unit. The study was approved by the Ethics Committee of the First Affiliated Hospital of Xinjiang Medical University (Ethics number: 20210226−168). All participants provided voluntary written informed consent. Information obtained only from those volunteers who were willing to participate could lead to selection bias. The inclusion criteria were: (1) have a normal sex life without use of contraceptive measures; the woman partner had not become naturally pregnant within one year due to men infertility; the woman partner had regular menstruation; a gynaecological examination did not show obvious reproductive abnormalities; and the potential participant was able to read and understand the content of questionnaire and complete it independently. (2) Routine examination of semen found abnormalities (according to the sixth edition of the World Health Organization semen analysis standards [18]); and the patient provided voluntary informed consent to participate in the study.

The exclusion criteria were: (1) serious illness (serious mental illness, serious chronic disease, or cancer); (2) psychological intervention experience in the past six months; (3) difficulty understanding the contents of the questionnaire or inability to complete the questionnaire.

### Measurement methods

The scales used in this study were all internationally available questionnaires so that results were comparable with other studies globally; therefore, no adjustments were made to these scales.

The Fertility Problem Inventory (FPI) consists of 46 items and contains five subscales, namely, social relationship (10 items), sexual relationships (8 items), parental role needs (10 items), marital relationships (10 items), and childless lifestyles (8 items) [19]. Each item was scored on a 6-point Likert scale, ranging from ‘completely disagree’ (1 point) to ‘completely agree’ (6 points). The total score ranged from 46 to 276 points. Higher scores were indicative of greater fertility pressure. The total FPI score and each subscale have been shown to have high reliability and validity, with Cronbach’s α values ranging from 0.77 to 0.93.

The Fertility Quality of Life (FertiQol) is a self-rated questionnaire designed by representatives from the European Society of Human Reproduction and Embryology and the American Society of Reproductive Medicine to accurately assess the quality of life of patients with infertility [20]. The FertiQol scale contains 36 items, including two independent items, and the remaining 34 items are divided between two subdimensions: one core dimension and one treatment dimension. The core dimension consists of four parts: emotional response (6 items), physical and mental relationship (6 items), marital relationship (6 items), and social relationship (6 items). The therapeutic dimension consists of two parts: environment (6 items) and tolerance (4 items). The score value of each option ranges from 0 to 4 points. Each subscale and the total scale can be converted to 0–100 points by calculation. Higher score values indicate a better quality of reproductive life. The Chinese version of the scale has Cronbach’s α coefficient of 0.827.

The Social Support Rating Scale (SSRS), developed by Xiao et al. [21] from 1986 to 1993, is used to assess patients’ social support, including subjective support (individual experience or emotional support) and objective support (visible or actual support). The scale consists of three dimensions, including direct material assistance, presence and participation in group relationships, and support availability (individuals’ active use of various social supports, including ways of talking, ways of asking for help, and participation in activities). Of the 10 items, Items 1–4 and 8–10 are multiple choice questions, each with four options, scored as 1, 2, 3 or 4 points, respectively. Item 5 is scored as A, B, C and D, and each item is scored on a 4-point scale, ranging from none (1) to full support (4). Items 6 and 7 are counted as 0 points if the answer is ‘no source.’ The total SSRS score of social support was calculated. Higher scores indicated a higher degree of social support.

The Mindful Attention Awareness Scale (MAAS), developed by Brown and Ryan [22], measures levels of mindfulness based on the concept of ‘current attention and awareness.’ The MAAS is a single-dimensional structure consisting of 15 questions related to cognitive, emotional, physiological, and other aspects of daily life. The 6-point Likert scale (with 1 indicating ‘almost always’ and 6 indicating ‘never’) has a minimum score of 15 and a maximum score of 90, with higher scores representing higher mindfulness levels.

### Covariates

Behavioural and sociodemographic data, including age, ethnicity, place of residence, monthly income, level of education, smoking, alcohol consumption, sleep, exercise, and marital status, were provided by the participants. Clinical characteristics, fertility history, and fertility status were extracted from the participants’ electronic questionnaires.

### Statistical analysis

First, quantitative data were tested for normality, with P > 0.05 indicating a normal distribution, described by $\overset{―}{X}\pm SD$; otherwise, median and interquartile spacing were used to describe non-normally distributed data. Frequencies and percentages were used to describe the demographic and clinical variables. One-way ANOVA and t-test were used to investigate whether different groups of demographic factors had a significant effect on the study variables, and after the ANOVA revealed significant differences between groups, a post hoc t-test (PT) was used to further explore the variability that existed between specific groups. Quantitative description of the closeness of the linear relationship between the two study variables was carried out using Person’s correlation, which examined the bivariate correlations of fertility stress with social support, quality of reproductive life and its two sub-dimensions. We also used SPSS 26.0 (Process_v 4.0) to examine the relationship between the two moderating variables (social support and mindfulness) and reproductive quality of life and its two dimensions. Age and monthly income were selected as prior covariates because of their published associations with reproductive quality of life. In the case of significant interactions, we used a simple slope analysis, which allowed the identification of modulators (social support and mindfulness), reproductive stress, and reproductive quality of life. Significant interactions were plotted at 1 SD above and 1 SD below the mean of a particular regulator. Two-tailed P values < 0.05 were interpreted as statistically significant.

## Results

### Demographic characteristics

The participants’ demographic characteristics are shown in Table 1. A total of 469 men were enrolled, with a mean age of 32 years; the majority (83.4%) were from urban areas (83.4%), 39.3% had a bachelor’s degree or above, 88.3% had no reproductive history; 218 (46.5%) were smokers, and 251 (53.5%) were nonsmokers. The modal monthly household income was 4,000–6,999 yuan, with 194 participants (41.1%) in this income category.

**Table 1:** Demographic characteristics of the patients.

Variant	N(%)
**men gender**	469 (100)
**Age *(X ± *SD)**	32.42 ± 5.05 (19 ~ 59)
**Nation**	
Han nationality	358(76.3)
National minority	111(23.7)
**Residency**	
City	391 (83.4)
Rural	78 (16.6)
**Education**	
Junior high school and below	64 (13.6)
High school/secondary	96 (20.5)
junior college	125 (26.7)
undergraduate	165 (35.2)
Master’s degree and above	19 (4.1)
**Monthly family income**	
Less than 4,000 yuan	92 (19.6)
4000 ~ 6999 yuan	194 (41.1)
7000 ~ 9999 yuan	106 (22.6)
More than 10,000 yuan	77 (16.4)
**Smoking**	
Yes	218 (46.5)
No	251 (53.5)
**Daily alcohol consumption**	
>25g	29 (6.2)
≤25g	124 (26.4)
No alcohol	316 (67.4)
**Exercise**	
Never or occasionally	235 (50.1)
1-2 times/week	162 (34.5)
3-4 times/week	49 (10.4)
≥5 times/week	23 (4.9)
**Average sleep per night**	
<4 hours	2 (0.4)
4 ~ 6 hours	74 (15.8)
6 ~ 8 hours	337 (71.9)
>8 hours	56 (11.9)
**Marital status**	
Unmarried	15 (3.2)
Married	450 (95.9)
Divorcee	4 (0.9)
**Reproductive history**	
None	414 (88.3)
firstborn	47 (10)
second pregnancy	8 (1.7)

### Correlation between variables

In the analysis of bivariate associations, residence and marital status were the only demographic variables significantly correlated with fertility pressure (residence: *t* = −3.197, *P *= 0.002; marital status: *F *= 8.466, *P *< 0.001). Participants living in urban areas had lower fertility pressure than mens living in rural areas. Post-hoc t-test analysis showed significant group differences between divorced men and married and unmarried men (*P *= 0.001; and *P *< 0.001, respectively). Divorced participants reported greater stress regarding having children. Marital status (*F* = 7.745, *P *< 0.001), exercise (*F* = 5.184, *P *= 0.002), and reproductive history (*F* = 4.671, *P *= 0.010) were significantly associated with perceived social support. The intergroup comparison showed that married men participants had higher social support than divorced participants (*P *= 0.002), participants who never exercised had lower perceived social support than those who exercised at least three times a week (*P *= 0.001), and childless participants had lower perceived social support than those who had a second child (*P *= 0.019). Marital status (*F* = 5.562, *P* = 0.004), sleep (*F* =* *4.842, *P *= 0.003), and reproductive history (*F* = 4.807, *P *= 0.009) were significantly correlated with reproductive quality of life. Post-facto analysis found that married participants had a higher reproductive quality of life than divorced participants (*P *= 0.044). Participants who had slept less than 4 hours per night in the past 3 months had a lower reproductive quality of life than those who slept 6 ~ 8 hours and over 8 hours per night (*P* = 0.015; *P *= 0.011). There were no significant correlations between the demographic variables and mindfulness.

Reproductive stress was significantly negatively associated with reproductive quality of life (*r* = −0.106, *P *< 0.001) and core dimensions (*r* = −0.132, *P *< 0.001). Mindfulness and reproductive quality of life (*r* = 0.369, *P *< 0.001) and the two subdimensions ([Core dimensions]: *r* = 0.339, *P *< 0.001; [Therapeutic dimensions]: *r *= 0.322, *P *< 0.001) were significantly positively correlated, and social support was significantly negatively correlated with fertility pressure (*r* = −0.123, *P *< 0.001). Fertility quality of life (*r *= 0.377, *P *< 0.001) and its two subdimensions ([Core dimensions]: *r* = 0.337, *P *< 0.001; [Therapeutic dimensions] *r* = 0.306, *P *< 0.001) were significantly positively correlated with social support. The mean values, standard deviations, and correlation coefficients of the study variables are shown in Table 2.

**Table 2:** Correlation between variables.

Variable	$\begin{array}{c} \overset{―}{X}\pm SD \end{array}$	Social Support	Fertility Pressure	FertiQoL	Core- FertiQoL	Therapeutic -FertiQoL
MAAS	62.09 ± 16.10	0.286**	0.053	0.369**	0.339**	0.322**
Social Support	42.25 ± 7.91		−0.123**	0.377**	0.337**	0.306**
Fertility Pressure	165.98 ± 36.78			−0.106*	−0.132**	0.010
FertiQoL	69.13 ± 13.49				0.968**	0.796**
Core-FertiQoL	71.75 ± 15.47					0.631**
Therapeutic -FertiQoL	65.91 ± 14.91					

MAAS, Mindful Attention Awareness Scale; FertiQoL, Fertility Quality of Life; Core-FertiQoL, Core Dimension of Fertility Quality of Life; Treatment -FertiQoL, Treatment Dimension of Fertility Quality of Life; SD, Standard Deviation.

### Moderation models

All models included the covariates of age, ethnicity, place of residence, education, monthly income, marital status, reproductive history, smoking, drinking, sleep, and exercise.

#### Regulation of reproductive stress and quality of life through social support and mindfulness.

**Fig 1:**
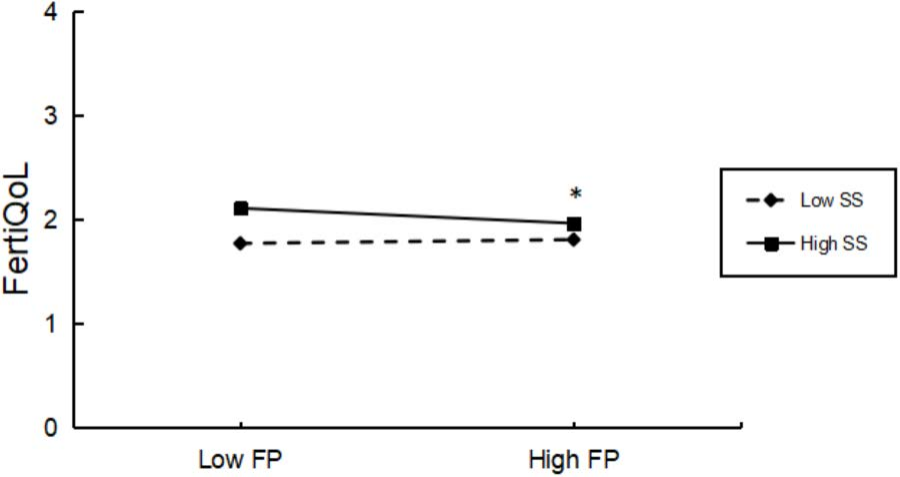
Interaction between social support and fertility pressure on fertiQoL graphed at one standard deviation above the mean (low) and one standard deviation above the mean (high). Abbreviations: FP, Fertility Pressure; SS, Social Support; FertiQOL, Fertility Quality of Life. * denotes p < 0.05.

Social support significantly moderated the association between reproductive stress and reproductive quality of life (*F* = 11.006, *P* = 0.001). Simple slope analysis showed that in men with higher social support (mean + 1 SD), reproductive stress was significantly associated with reproductive quality of life (*t* = −3.146, *P* = 0.002). However, this association was not significant in men with low social support (mean – 1 SD) (*t* = 0.906, *P* = 0.365). The moderating effect of social support is shown in Fig 1. Mindfulness did not have a significant moderating effect on the relationship between reproductive stress and reproductive quality of life (*F* = 1.528, *P* = 0.217).

#### Regulation of reproductive stress and its core dimensions through social support and mindfulness.

**Fig 2:**
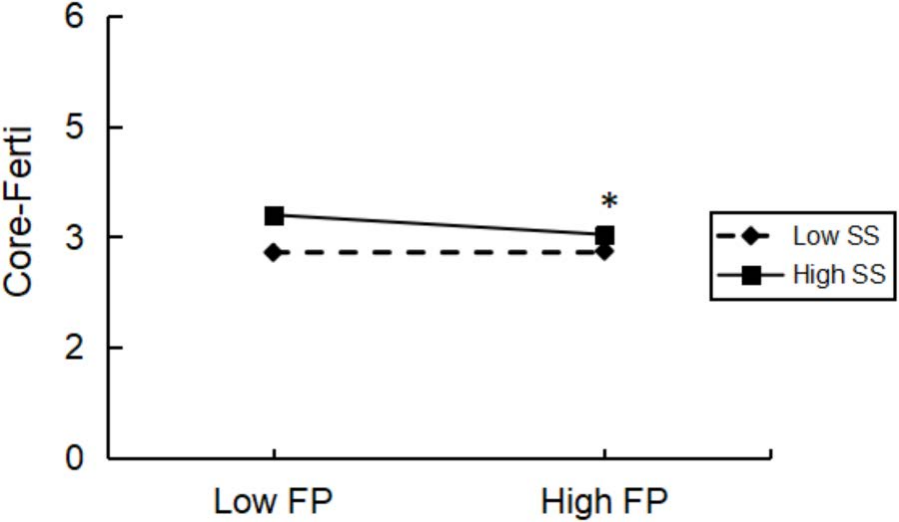
Interaction between social support and fertility pressure on core-ferti graphed at one standard deviation above the mean (low) and one standard deviation above the mean (high). Abbreviations: Core-Ferti, Core Dimensions of Fertility Quality of Life. FP, Fertility Pressure; SS, Social Support; FertiQOL, Fertility Quality of Life. * denotes p < 0.05.

Social support significantly moderated the relationship between fertility pressure and the core dimension (*F* = 9.063, *P* = 0.003). This association between fertility pressure and the core dimension was more marked in men with higher social support (mean + 1 SD) (*t* = −3.333, *P* = 0.001) than in men with low social support (mean – 1 SD), (*t* = 0.266, *P* = 0.790). Fig 2 shows the buffering effects of social support. However, mindfulness (*F* = 1.406, *P* = 0.236) did not affect this correlation.

#### Social support and mindfulness modulated fertility stress and its therapeutic dimensions.

**Fig 3:**
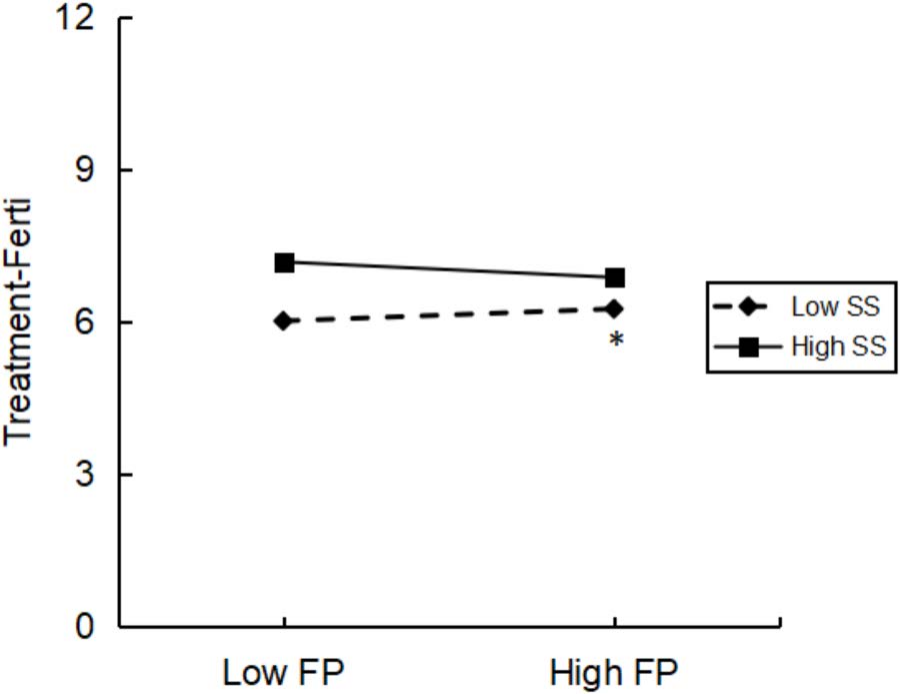
Interaction between social support and fertility pressure on treatment-ferti graphed at one standard deviation above the mean (low) and one standard deviation above the mean (high). Abbreviations: Treatment-Ferti, Therapeutic Dimension of Fertility Quality of Life. FP, Fertility Pressure; SS, Social Support; FertiQOL, Fertility Quality of Life. * denotes p < 0.05.

Although the bivariate association between fertility stress and the treatment dimension was not significant, social support significantly moderated this association (*F* = 9.383, *P* = 0.002), and interaction detection revealed a significant association between fertility stress and the treatment dimension in men with low social support (*t* = 2.531, *P* = 0.012). However, this association was not significant in men with higher social support (*t* = −1.448, *P* = 0.148). The moderating effect of social support is shown in Fig 3. There was no evidence that mindfulness modulated this association (*F *= 1.026, *P *= 0.312).

## Discussion

This cross-sectional analysis aimed to explore the mediating role of social support and mindfulness on the association between perceived reproductive stress and reproductive quality of life in infertile men. Previous studies, including a study by Lei et al. [23], found that in men with infertility, fertility pressure was negatively correlated with the quality of reproductive life. In individuals with infertility, fertility pressure may lead to negative emotions such as anxiety, depression, and feelings of inferiority. Excessive fertility pressure can lead to stigma, and in severe cases, it can lead to separation from the family and society, thereby compromising quality of life.

We attempted to establish a regulatory model of social support and mindfulness. The study results were consistent with our hypothesis that in men with infertility, a high level of perceived or actual social support from family and friends can help to actively mobilise their internal resources to cope with fertility pressure and relieve the negative emotions of anxiety and depression when facing infertility-related problems. The findings on the moderating effect of social support are consistent with previous research, including a cross-sectional study that found that subjective social support plays a mediating role between negative life events and relapse in 215 patients with schizophrenia [24]. Additionally, social support, as a positive psychological resource, can effectively buffer the impact of adverse events on outcomes. PingHu’s [25] analysis of 202 men infertility patients showed a high prevalence of anxiety (67.8%). Structural equation modelling (SEM) found that self-efficacy and social support mediated the relationship between family functioning and anxiety, and that family functioning had a significant impact on the mental health of men infertility patients, with self-efficacy and social support being key mediators.

Our further study found that men with low social support showed a significant positive correlation between fertility pressure and the treatment dimension; that is, the lower the social support received, the stronger the positive correlation between fertility pressure and the treatment dimension. This finding differs from that of a previous study which found that fertility pressure was negatively correlated with reproductive quality of life and its two subdimensions [26]. Social support may play a regulating role, or gender differences may lead to a different relationship between fertility pressure and the treatment dimension in men and women. Previous studies focused on women with infertility, whereas this study focused on men with infertility. Previous studies have shown that women are more sensitive to the stressful infertility events, tend to focus on them, and think about them repeatedly. In addition, affected by the traditional Chinese idea that ‘there are three things that are unfilial; having no offspring is the greatest,’ infertile women often feel greater fertility pressure, resulting in poor treatment tolerance [19,27,28]. In contrast, men tend to adopt a more positive coping style in the face of stressful events. The greater the stress experienced during the long course of treatment, the higher their treatment compliance and tolerance.

Mindfulness has also been shown to be a coping mechanism as well as a positive and variable psychological trait. Many foreign and domestic experts and scholars have confirmed that mindfulness training is conducive to improving physical and mental health [16,29–32]. Several studies have shown that mindfulness can be used as a regulatory factor [33–35]. For example, when Ma et al. [36] discussed the regulation of the relationship between personality characteristics and sleep quality in men and women with infertility, they found that mindfulness plays a regulatory role between neuroticism and sleep quality in women with infertility. In addition, Stella Snyder et al. [13] found that, in 56 patients with lung cancer, higher levels of mindfulness protected women from stigma and cancer-related symptoms. Using a case-control study, M·A’s path analysis of 250 infertile men compared with 500 healthy men found that positive thinking was an important factor in infertile men’s quality of reproductive life, and that increasing levels of positive thinking improved their confidence in clinical diagnosis and infertility treatment, enabling them to cope positively with these challenges [37]. In this study, however, mindfulness was not a significant buffer against fertility stress. Mindfulness may therefore be an inadequate protective factor. The possible causes could be that the sample was highly homogeneous and there was little variation in the level of mindfulness among the patients in the sample, resulting in a non-significant moderating effect. Alternatively, the moderating effect of mindfulness was weak and the direct effect of fertility stress on the quality of reproductive life may have been too strong, masking the moderating effect of mindfulness. Future studies should increase the sample size to ensure that there is sufficient variability in the level of mindfulness among the patients in the sample. The results suggest that higher social support levels may protect people with infertility from the negative effects of fertility stress on reproductive quality of life. Social support may interrupt negative thoughts such as anxiety and depression caused by fertility pressure, which suggests that medical staff and family members should provide more care and support to men patients with infertility to prevent a decline in the patient’s reproductive quality of life caused by serious fertility pressure.

Chinese collectivist culture has a unique impact on the social support and positive outcomes of men infertility patients. In traditional Chinese culture, reproduction is seen as the core of family responsibility, and men, in particular, bear the important mission of ‘transmitting the family lineage’; these cultural concepts may make it more difficult for men patients to receive social support, and family members may exert pressure on infertility patients to solve their problems as soon as possible. This may cause patients to focus more on the impact of infertility on the family and neglect their own psychological needs, resulting in ambivalence. Regarding support from outside the family, ‘face’ is an important social concept in Chinese culture. Infertility may be seen as a ‘loss of face’and these patients may be concerned about the stigma attached to infertility. men patients may be reluctant to seek support from outside the family (e.g., friends or professional counselling), which may lead to feelings of isolation and helplessness. However, positive thinking exercises can help patients accept their current situation in a non-judgemental way, reduce reliance on traditional masculinity, and reduce excessive concern for ‘face’. Thus, alleviating negative emotions brought about by cultural pressures, and helping patients to face their emotions openly according to Chinese culture, and incorporating traditional cultural elements (e.g., Tai Chi, meditation) to design positive thinking programmes, while encouraging the participation of family members to enhance the effectiveness of social support.

At present, China is encouraging moderate fertility by implementing a ‘three-child policy’, which allows each couple to have three children. Although, the fertility policy may reduce the pressure on some families to have children, the economic and social pressures may still have an impact on mental health, and the cost and time invested in childcare may have an impact on a family’s quality of life, especially for low- and middle-income families. Therefore, the implementation of the fertility policy has little effect, in this case, to solve the ‘men’ infertility problem seems to be the accelerator of the policy. Considering the progress of medical technology, solving the physical level is not enough to improve the cure rate of infertility, but paying attention to the psychological needs of men infertility patients is of great public health importance in treating infertility and improving the quality of reproductive life.

This study has some limitations. First, the samples were designed for a single centre and lacked versatility. Future studies should include multiple hospitals and communities to promote health equity. Second, the use of self-reported data by patients is prone to potential bias, as well as possible recall bias, and in the future, respondent information should be collected on a regular basis to reduce potential bias. Finally, this was a cross-sectional study, and although we could assess the associations, we are unable to make causal inferences. Further longitudinal studies are needed to reveal cause and effect.

## Conclusion

Perceived fertility pressure may be associated with a reduced reproductive quality of life in men with infertility. Social support appears to moderate this link. Therefore, a high level of social support may have a protective effect. Healthcare providers and family members should be encouraged to interact with patients with infertility at multiple levels and provide support and care.
